# Functional Rehabilitation of Anterior Cruciate Ligament Tear in the Pediatric Population: A Comprehensive Review

**DOI:** 10.7759/cureus.49863

**Published:** 2023-12-03

**Authors:** Khushi Khurana, Gajanan Pisulkar

**Affiliations:** 1 Orthopaedics and Rehabilitation, Jawaharlal Nehru Medical College, Datta Meghe Institute of Higher Education and Research, Wardha, IND; 2 Orthopaedic Surgery, Jawaharlal Nehru Medical College, Datta Meghe Institute of Higher Education and Research, Wardha, IND

**Keywords:** return to sport, reconstruction surgery, rehabilitation, pediatric, acl tear

## Abstract

Pediatric sports injuries are a growing concern due to increased youth participation in sports. Effective rehabilitation strategies are essential for ensuring optimal recovery, restoring knee function, and preventing long-term consequences. This research aims to explore and evaluate various functional rehabilitation approaches tailored to pediatric anterior cruciate ligament (ACL) tear injuries. Functional rehabilitation of ACL tears in pediatric sports injuries is an important area of research due to the unique considerations and challenges that arise when treating ACL injuries in young athletes. Over the last 20 years, there has been a well-documented uptick in ACL injuries among pediatric populations. This rise can be attributed to the growing involvement of the younger population in competitive sports, as well as heightened awareness regarding sports-linked injuries. This study highlights the importance of early surgical reconstruction in children to enable a quick return to sports and prevent long-term cartilage and meniscal damage resulting from instability. The use of physeal-sparing ACL reconstruction techniques, particularly hamstring autografts, is recommended for favorable clinical outcomes while minimizing growth disturbances. This study offers valuable insights for healthcare professionals and researchers, serving as a reference to guide optimal approaches in managing pediatric ACL injuries and achieving successful results in this field.

## Introduction and background

Over the past two decades, there has been a notable and gradual rise in documented cases of pediatric anterior cruciate ligament (ACL) injuries, driven by various factors [1]. Non-surgical methods are a viable choice for addressing ACL injuries in children. These approaches involve physical therapy routines designed to enhance neuromuscular control. These exercises are implemented across various stages of the recovery process [1]. Contemporary literature leans toward advocating for surgical intervention, even among children, due to its effectiveness in mitigating the risk of concurrent meniscal injuries and chondral injuries [2-6]. Albeit, a notable concern pertains to potential physeal injuries arising from surgical procedures. The most frequent associated injuries include a specific pattern of bone bruising that does not extend into the metaphysis, as well as lateral meniscus tears. This challenge has been addressed through refined techniques in anterior cruciate ligament reconstruction (ACLR) [6-8].

Ardern et al. in 2018 authored an article that was a collaborative effort involving a global cohort of orthopedic surgeons and physiotherapists who collectively addressed six fundamental clinical queries concerning pediatric ACL injuries. The resultant consensus statement serves as a comprehensive guide to aid medical practitioners, provide support for children afflicted with ACL injuries, and assist parents in making well-informed decisions. Subsequently, several original articles have delved into the realm of setbacks following pediatric ACLR, encompassing aspects such as contralateral ACL injuries and graft rupture, along with assessing outcomes after quadriceps tendon (QT) autograft reconstruction [9].

There is not a perfect graft option for pediatric patients overall, with each associated with its own advantages and drawbacks. It is crucial to recognize that there is no one-size-fits-all graft for any specific patient. Instead, we aim to present the different choices available, drawing from the most recent data, scientific insights, and our combined expertise to illustrate these options. This work endeavors to furnish an all-encompassing review of the literature from the past decade. It particularly focuses on various facets, including incidence rates, mechanisms of injury, the evolutionary trajectory of treatment methodologies, graft selection, related intra-articular injuries, as well as the spectrum of complications.

## Review

Methodology

**Figure 1:**
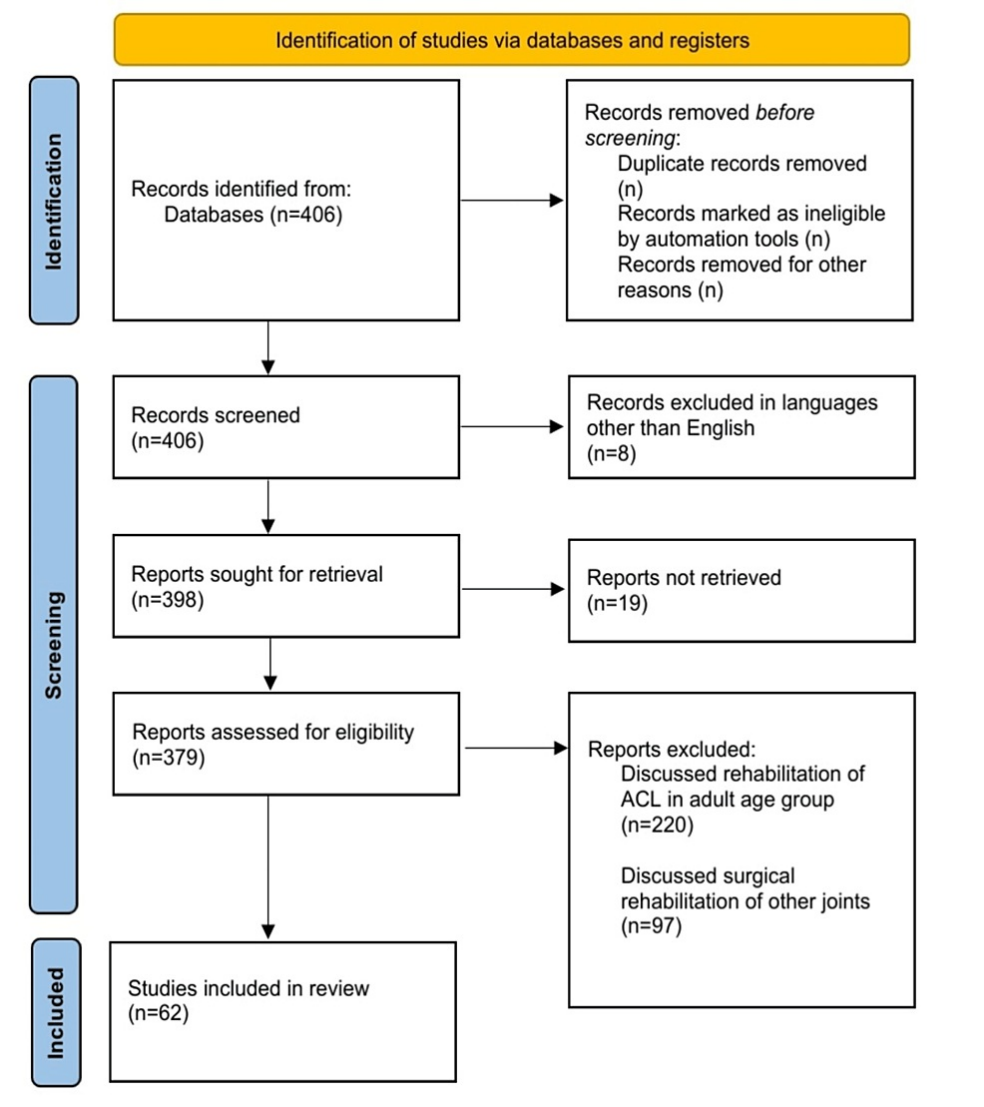
PRISMA flow diagram for screening and selecting articles. PRISMA: Preferred Reporting Items for Systematic Reviews and Meta-Analyses; ACL: anterior cruciate ligament

We searched PubMed, Scopus, and Google Scholar databases to identify common sports injuries, existing rehabilitation protocols for ACL injuries in the pediatric age group, and gaps in the current approaches. We used certain keywords, i.e., “ACL tear,” “Pediatric”, “reconstruction”, “return to sports,” and “rehabilitation.” Ultimately, the articles that presented novel insights were selected for inclusion in this review. We excluded articles that were not retrievable or discussed the rehabilitation of ACL in adults and rehabilitation of other joints. The review was performed following predetermined research inquiries. Of the 406 articles screened , 62 were found to be relevant and included. Figure 1 depicts the Preferred Reporting Items for Systematic Reviews and Meta-Analyses (PRISMA) flowchart for the literature search.

The literature unveils that the predominant mechanism for pediatric ACL injuries (constituting 71%) is non-contact in nature. This is likely attributed to the strain induced during pivoting movements, where the knee is semi-flexed and the foot is in contact with the ground [10,11]. Contemporary evidence underscores that non-contact ACL ruptures arise from neuromuscular and biomechanical factors during dynamic motions. Nevertheless, the precise movements and the extent of neuromuscular control within the pediatric patient cohort remain inadequately understood [12,13]. An additional mode of pediatric ACL injury involves hyperextension accompanied by either valgus or rotational forces [9].

In comparison to adults, children and adolescents demonstrate a notably higher prevalence of partial ACL tears relative to complete tears [14]. Furthermore, ACL enthesis tears, with the distal region being more commonly affected than the proximal, have been observed among adolescents [15]. Notably, the tibial eminence in the pediatric age group does not achieve full ossification, rendering the bone beneath more prone to failing in contrast to the robust ACL [16]. This structural characteristic renders the pliable intercondylar eminence of the tibia susceptible to avulsion caused by traction in the junction between cartilage and bone [17,18]. Nonetheless, a varied degree of plastic alterations of the fibers of the ACL might also occur, leading to persistent clinical laxity even after the anatomical alignment of the fracture has been restored [19-21].

Pediatric ACL injuries can manifest as tibial spine avulsions or ACL tears. Traditional approaches included old-fashioned measures such as casting, bracing, improvements in activities, and physical therapy [22-24]. However, contemporary evidence leans toward early surgical intervention due to documented subpar outcomes in conservatively managed cases, often leading to prolonged instability and an elevated risk of chondral damage and meniscal injury [25-27]. Some non-operatively treated young athletes manage to resume sports either equivalent or sometimes at a greater level compared to those who undergo surgical reconstruction [28,29].

Advocating for injury prevention and implementing prevention programs for skeletally immature athletes are recommended [13]. Non-operative management is presently restricted to instances of partial tears of ACL with a negative pivot shift test, type one fractures of tibial eminence, and type two fractures with minimal displacements [28]. This approach encompasses robust rehabilitation and utilization of braces that are protective during rigorous physical activities. It serves as a secure and workable route for patients devoid of accompanying injuries or notable instability issues.

Reconstruction and graft materials

Surgical reconstruction becomes imperative in pediatric patients when a ruptured ACL is linked to a positive pivot shift test. The prevailing consensus among most surgeons is that ACLR should be undertaken after achieving an improved knee range of motion (ROM) except if circumstances involve a tibial eminence fracture or a concurrent bucket-handle meniscus injury [9,28,30].

Regarding ACLR in pediatric and adolescent populations, a few primary ways are presently employed, namely, a collective extra and intra-articular reconstruction of the iliotibial band, a transphyseal ACLR, an all-epiphyseal ACLR, an integrated approach involving a transphyseal tunnel in the tibia, and all-epiphyseal tunnel in femur [22,24,31-35]. Primary considerations for determining the appropriate approach should encompass factors such as age, height, Tanner stage (which indicates the degree of sexual maturation), and the surgeon’s expertise and training. These considerations play a pivotal role in guiding the choice of approach best suited for each case [35].

The anatomic transtibial technique for ACLR offers options of single-bundle reconstruction or a double-bundle reconstruction, with the primary benefit of restoring natural knee kinematics. Lawrence et al. introduced an alternative approach, an all-epiphyseal technique, which is a modification of Allen F Anderson’s original method [14,36]. This technique involves the creation of a lateral femoral epiphyseal tunnel and an oblique tibial epiphyseal tunnel, carefully protecting the physis (growth plate). The graft is secured within these tunnels using bio-absorbable screws. By circumventing the physis, this method minimizes the risk of disturbing the growth, limb length inequalities, and angular deformities. However, it is important to note that the acute angles formed by these tunnels can increase strain on the graft, potentially leading to a heightened chance of graft failure [14].

More recently, Pennock et al. made modifications to the all-epiphyseal technique for ACLR in the pediatric population. In this adaptation, graft stabilization involves a suspensory fixation in the tibia and the use of the interference screw on the femur. This innovation eliminates the use of sutures over the physis, preventing inadvertent restriction of the physis [37]. Furthermore, the stability achieved is absolutely anatomical, and both tunnels are meticulously positioned at the center of the ACL’s footprint. The stability achieved through suspensory fixation and interference screw might even surpass that of post-suturing or fixation to the periosteum [37].

In hybrid ACLR, the creation of a tunnel across the tibial physis proximally can disrupt the growth plate. The graft is affixed to the lateral femur, preserving the distal femur physis, which contributes more significantly to lower limb growth. Mall and Paletta introduced a modified technique for transphyseal ACLR, securing the femoral area with a suspensory device and using a stapler, screws and post, or suspensory device for tibial stabilization [28]. Willson et al. reported precocious outcomes in pediatric athletes with an average bone age of 12 years implementing a hybrid technique involving a tibial tunnel transphyseally while constraining the femoral physis. In their study, 91% (21 out of 23) of cases maintained equal limb lengths during an average 21-month tracking. They employed an adjustable loop cortical suspension device for the fixation of the femur and various methods such as an interference screw, cortical button, metaphyseal screws and plates, or staples for the fixation of the tibia [38].

When dealing with tibial eminence fractures, fixation methods can include both open surgery and arthroscopy. Fixation options encompass metal screws, sutures, suture anchors, Kirschner wires, or bio-absorbable nails. Suture fixation is preferred due to concerns over screw-induced comminution or weakening of the small fragmentary parts. Sutures have comparable or even better strength characteristics [39-41]. However, in cases of large, non-comminuted avulsed fragments, we can consider screw fixation.

Regarding ACL repair, van Eck et al. conducted a systematic review suggesting that primary repair is more successful in cases of proximal ACL fiber tears compared to tears that are mid-substance or present distally. They determined that repair of an ACL tear could be a viable choice, especially for patients experiencing acute ACL tears proximally, particularly in individuals who are skeletally not mature [42].

Another approach involves internal bracing. Direct repair was combined with internal bracing in a few cases of ACL tears with tibial spine fractures by Smith et al. A follow-up at three months demonstrated complete healing during second-look arthroscopy [43]. In addition to sutures, alternative augmentation methods such as patellar tendon, collagen scaffolds, or iliotibial bands have been proposed, potentially enhancing the likelihood of a successful repair [41].

In the early years of this century, the options of grafts for ACL reconstruction in the pediatric age group included hamstring tendon (HT) autograft, QT autograft, bone-patellar-tendon-bone (BPTB) autograft, iliotibial band, and several soft-tissue allografts [44,45]. While the spectrum of graft choices remains relatively constant, HT autografts have gained prominence as the preferred option globally. Nevertheless, recent investigations have tilted the balance toward QT autografts over HT autografts, primarily due to a decreased incidence of graft ruptures associated with QT autografts [1,44-46]. Table 1 depicts grafts used in ACLR.

**Table 1:** Grafts for anterior cruciate ligament tear reconstruction.

Anterior cruciate ligament tear reconstruction grafts
Hamstring tendon autograft
Quadriceps tendon autograft
Bone-patellar-tendon-bone autograft
Iliotibial band autograft
Soft-tissue allografts

Pennock et al. revealed a graft failure rate of 21% in the HT group compared to 4% in the QT group for transphyseal ACLR [44]. Runer et al. documented a 5.5-fold higher rate of revisions in children under 15 years of age than in adults over 45 years, and within the pediatric cohort, patients receiving HT autografts exhibited a 2.7-fold higher revision rate compared to those receiving QT autografts. The reduced graft rupture rate associated with QT autografts is attributed to their larger size and the sustained function of hamstrings, as they do not require hamstring harvesting. Even though the rate of graft rupture of QT autografts is comparable to those of BPTB autografts, certain factors such as anteriorly knee pain, a heightened likelihood of fracture of the patella, and rupture of patellar tendon mildly favor QT autografts compared to BPTB. Nonetheless, QT autografts are not devoid of complications, including initial extensor deficits, arthrofibrosis, and a longer recovery period postoperatively. Presently, the existing data lacks definitive high-level proof that decisively favors QT over HT autografts. Going forward, the need of the hour is a comprehensive study conducted at multiple centers with an extended follow-up that can provide higher-level evidence for the comparison of QT and HT autografts, thus guiding the optimal choices of grafts for pediatric ACLRs [45].

Disturbance in the growth plate

Growth disturbances present a concern as physeal arrest can occur as a result of injury to the physis when employing the transphyseal technique involving drilling across the physis. The size of the tunnel and the angle at which it is drilled are pivotal factors contributing to physeal injury, along with elevated graft tensions. To mitigate risks, it is recommended to employ tunnels with smaller diameters and soft-tissue autografts. It is also advised to orient the tunnel vertically, minimize graft tension, and limit hardware placement across the lateral distal femoral physis [27].

A study by Fauno et al. investigated 39 children who underwent transphyseal ACLR while their growth plates were still open [47]. They discovered that 24% of the children exhibited minute limb length discrepancies at osseous development. They reported that the deformity in the valgus angulation induced surgically in the distal femur was typically compensated for by a varus deformation in the tibia proximally [47].

Wong et al. conducted a meta-analysis of 45 studies involving 1,321 patients and 1,392 knees. They reported that 58 growth disturbances were documented. Among these, corrective surgeries were needed for 16, i.e., 27%. The analysis revealed that angular deformities were experienced by almost 4%, mostly valgus, and 7% exhibited limb length disturbances of at least 1 cm [48].

Other injuries

ACL tears in skeletally growing patients often coincide with additional injuries such as meniscal damage, bone impact injuries, and chondral damage. Bordoni et al. conducted an MRI study that revealed various associated injuries in pediatric patients, exhibiting contusions in bones, meniscal damage, injuries of other ligaments, cartilage defects, and associated fractured patella. Notably, bone bruise distribution and affected areas were found to be similar between adults and children; however, bone bruises linked to ACL tears seemed to be less frequent in pediatric cases compared to adults [49].

In a different MRI study by Novaretti et al., a significant difference was noted in bone contusions that extended from the physis into the metaphysis between skeletally immature and skeletally mature groups of patients. Pediatric patients with ACL injuries exhibited a distinct bone bruise pattern when compared to those with mature skeletons, with greater metaphyseal involvement. The most common regions affected in the pediatric age group were the medial or the lateral and central regions of the tibia, as well as the lateral region of the femur without metaphyseal extension [50].

A multicenter study conducted by Feucht et al. on tibial eminence fractures found that more than a quarter of children who underwent surgery had arthroscopically confirmed meniscal injuries. Among these cases, 90% involved lateral meniscal injuries, with longitudinal tears in the posterior horn being the most prevalent. Other sites included the anterior horn of the lateral meniscus at the site of root detachment [51].

A retrospective analysis by Vavken et al. encompassing 208 skeletally immature patients who underwent ACLR indicated that almost 35% had lateral meniscal tears, almost a similar percentage of patients had medial meniscal tears, and 5% had chondral lesions. They also found a connection between meniscal tears and delayed surgical intervention, with body mass index and the interval between ACL tear and surgical intervention serving as criteria for investigating cartilage damage [52].

Malatray et al. determined that the incidence of ACL-assisted lesions around the menisci in the skeletally immature population is comparable to adults. They recommend systematic assessment through the intercondylar notch during ACLR for precise diagnosis [53]. A study by Rhodes et al. assessed the correlation between findings of MRI and surgical observations in patients with avulsion of the tibial spine, noting higher evidence of entrapment of meniscus during surgery when compared to MRI findings. The most commonly undetected meniscal damage was in the posterior horn, notably a vertical tear [54].

Kinsella et al. examined accompanied posterolateral corner injuries in skeletally immature patients with ACL injuries, finding that nearly half of the patients had a related posterolateral corner injury, and about 14% had a complete tear. Age was correlated with posterolateral corner injury, with an increase in the damage to the posterolateral corner by nearly 1.8 times with each additional year of age. No correlation was noted between posterolateral corner injury and ACL graft failure [55].

In summary, the data underscore that the most frequently associated injuries are bone bruises that do not extend into the metaphysis and lateral meniscus injuries.

Return to sports

Return to sports (RTS) following primary ACLR in children and adolescents demands careful consideration due to the elevated risk of graft rupture in this population compared to adults [56]. Various studies have shed light on the optimal timing and criteria for granting RTS clearance to young athletes.

Paterno et al. identified factors predictive of a secondary ACL tear following initial ACLR and RTS. Susceptible individuals were identified based on age, gender, triple hop performance, and knee-related confidence. This vulnerable group was five times more likely to sustain a secondary ACL tear [57].

Cordasco et al. categorized athletes under 20 into three groups based on ACLR type. Middle-school-aged athletes who received transphyseal reconstruction with hamstring autograft had higher rates of revision and lower rates of RTS than other groups. This suggests that this group with intertwined risk profiles should be incorporated into preoperative counseling regarding surgical expectations and RTS rates [58]. Beischer et al. compared adolescents to adults after primary ACLR. Most adolescents returned to sports sooner than adults but without wholly recovering functions of the muscles. They advised clinicians to caution young athletes against early RTS until fully functional muscles, which may require about 12 months [59].

A multicenter study comparing pediatric open physis patients to skeletally mature groups was conducted by Geffroy et al., regarding RTS after prior ACLR. In the open physis group, return to contact sports was advised to be delayed until 14 months after surgery to mitigate the risk of re-injury [60]. Ithurburn et al. reported that following all-epiphyseal physeal-sparing ACLR, pediatric athletes exhibited superior quadriceps femoris strength symmetry, elevated self-reported knee function, and higher performance of knee function compared to groups with closed physes after conventional ACLR [61].

In summary, RTS after pediatric ACLR should be approached cautiously. Delaying RTS until muscle function is fully regained, adopting appropriate risk assessment, and understanding age-specific risk profiles can aid in making informed decisions that minimize the likelihood of graft rupture and secondary ACL injuries [62]. Table 2 presents a summary.

**Table 2:** Summary table. ACL: anterior cruciate ligament; ACLR: anterior cruciate ligament reconstruction; RTS: return to sports

Authors	Summary
Mark et al., 2023 [1]	The chances of undergoing growth-related issues after an ACLR are lower in skeletally immature patients when compared to adults
Beck et al., 2017 [2]	Over the past two decades, there has been a notable rise in the occurrence of ACL tears among pediatric patients
Airchroth et al., 2002 [3]	Only conservative treatment of ACL tear may lead to instability of the knee
Ramski et al., 2014 [4]	Patients who received delayed treatment exhibited abnormal joint laxity when compared to those who underwent early operative stabilization
Werner et al., 2016 [5]	There has been notable rise in frequency of concurrent meniscal and cartilage procedures among pediatric patients undergoing ACLR
Dumont et al., 2012 [6]	Elevated lateral and medial meniscal tear has been linked to ACL injuries
Kocher et al., 2002 [7]	There is a significant concern regarding the possibility of physeal injuries resulting from surgical procedures
Bonnard et al., 2011 [8]	Physeal-sparing reconstruction has shown better functional results and has lower rates of revision surgeries
Ardern et al., 2018 [9]	It offers a thorough evidence-based summary that assists healthcare providers in making the most well-considered decisions
Domzalski et al., 2010 [10]	The primary cause of pediatric ACL injuries is non-contact in nature
Yu et al., 2007 [11]	Stress is experienced when knees are partially bent and the foot is in contact with the ground, leading to ACL injury
Holden et al., 2016 [12]	ACL rupture results from neuromuscular and biomechanical factors during dynamic movements
Moksnes et al., 2016 [13]	Neuromuscular training helps in better rehabilitation
Stanitski et al., 1993 [14]	Partial tears are more common than complete tears in skeletally immature people
Xavier et al., 2016 [15]	Distal region is more frequently affected than the proximal region in tears in the ACL enthesis
Herring, 2002 [16]	In the pediatric age group, tibial eminence does not fully ossify
Lafrance et al., 2010 [17]	The flexible inetercondylar eminence of the tibia is vulnerable to avulsion
Shin, et al., 2015 [18]	Tibial eminence fractures occurs due to traction between bone and cartilage
Kocher et al., 2003 [19]	Patients with tibial eminence fractures frequently experience meniscal entrapment
Tudisco et al., 2010 [20]	The prognosis is closely associated with fracture type, achieving anatomical reduction, and maintaining articular congruity
Biden et al., 2000 [21]	Different degrees of plastic change in the fibers of ACL occurs, resulting in clinical laxity
Anderson, 2004 [22]	ACL injuries in pediatric patients can present as tibial spine avulsions
Beynnon et al., 2005 [23]	ACL injuries make the knee more susceptible to future injuries and early development of osteoarthritis
Kocher et al., 2007 [24]	Standard method of ACL injury management includes casting and physical therapy and stabilization
Lawrence et al., 2011 [25]	A significant increase is noted in the prevalence of medial meniscal tear if ACLR is delayed for more than 12 weeks
Millett et al., 2002 [26]	Occurence of medial meniscal tear is highly associated with delayed surgical management
Trivedi et al., 2017 [27]	Early surgical measures result in better outcomes
Mall et al., 2013 [28]	Non-operative management is limited to cases of partial ACL tears
Aronowitz, 2000 [29]	In certain cases, non-operatively treated skeletally immature patients resume sports better than surgically treated ones
Shelbourne et al., 1991 [30]	Arthrofibrosis, a complication of ACLR hinders a patient’s utility to regain full range of motion
Kocher et al., 2005 [31]	The utilization of a physeal-sparing for ACLR in skeletally immature patients, employing an autologous iliotibial band graft, yields outstanding functional results
Micheli et al., 1999 [32]	A modified combination of intra and extra articular anterior cruciate ligament reconstruction can be used.
Fabricant et al., 2017 [33]	Physeal sparing ACLR have been devised for application in pediatric patients to reduce the likelihood of growth disturbances
Lo et al., 1997 [34]	The transphyseal way can be used for ACLR
Demange et al., 2014 [35]	Age, height, Tanner stage, and surgeon’s proficiency and training are the most suitable criteria for the selection of most appropriate approach
Lawrence et al., 2010 [36]	An all-epiphyseal technique has been used for ACLR, which is a modified Allen F Anderson’s original technique.
Pennock et al., 2018 [37]	All-epiphyseal ACLR modification involves use of suspensory fixation of tibia and interference screw in femur
Willson et al., 2018 [38]	For femur fixation, an adjustable loop cortical suspension device can be used
Berg, 1993 [39]	The absence of secure fixation and cautious rehabilitation plan led to the development of arthrofibrosis, restricting knee motion
Mahar et al., 2008 [40]	Both sutures as well as resorbable screw configurations lead to more deformation compared to that of resorbable nails or a metal screw
Bongo et al., 2005 [41]	Suture fixation is favored as sutures exhibit superior strength
Van Eck et al., 2018 [42]	When it comes to ACL tears , proximal tear patterns have demonstrated a higher likelihood of successful healing through primary repair compared to distal tears or mid substance tears
Smith et al., 2016 [43]	Inclusion of internal bracing has been found to enhance the rate of success of ACL repair
Pennock et al., 2019 [44]	ACLR in children can be effectively carried out using hamstring tendon autografts or quadriceps tendon autografts, resulting in favorable outcomes
Runer et al., 2020 [45]	Age groups, types of graft, and levels of activity determine the necessity of revision surgeries
Andernord et al., 2015 [46]	Adolescents faced a higher risk of requiring revision surgery following ACLR
Faunø et al., 2016 [47]	The distal femoral valves regulation created during surgery is offset by a proximal tibial varus angulation
Wong et al., 2019 [48]	Valgus is the most common angular deformity experienced by the patients
Bordoni et al., 2019 [49]	In pediatric cases, bone bruises associated with ACL tears appear to occur less frequently when compared to adults
Novaretti et al., 2018 [50]	Skeletally immature patients show different bone bruise pattern in comparison to those with fully developed skeletons
Feucht et al., 2017 [51]	It should be anticipated that meniscal injuries will occur in children undergoing surgical management for tibial eminence fracture
Vavken et al., 2018 [52]	Both lateral and medial meniscal injuries are found to be common in patients who undergo ACLR
Malatray et al., 2018 [53]	The occurrence of ramp lesions associated with ACL injuries in children and adolescents is comparable to what is observed in adult populations
Rhodes et al., 2018 [54]	The occurrence of meniscal entrapment discovered during surgery is found to be high despite the relatively low incidence of positive findings on MRI scan
Kinsella et al., 2019 [55]	This review highlights the frequency of posterolateral corner injuries in the context of simultaneous ACL injuries among pediatric patient, a distinct and unique population
Davies et al., 2020 [56]	Current evidence suggests that hop testing, used as measure to evaluate function after ACLR, lacks consistency in predicting successful rehab outcomes
Paterno et al., 2017 [57]	Individuals deemed vulnerable to experience a second ACL tear were based on age, gender, and triple hop performances
Codarsco et al., 2019 [58]	Middle-school aged category should undergo preoperative counseling discussions regarding surgical expectations and the likelihood of RTS
Beischer et al., 2018 [59]	Many adolescents resumed their sport activities earlier than adults, but without completely regaining full muscle functions
Geffroy et al., 2018 [60]	In skeletally immature groups, it takes significantly longer time to return to sports, often extending beyond one year. The return to competitive sports take even longer and are challenging
Ithurburn et al., 2019 [61]	When young athletes were cleared to RTS after ACLR, they exhibited greater symmetry in quadriceps strength and had better knee-related function compared to adolescents who had undergone conventional ACLR
Moloney et al., 2022 [62]	Postponing the return until full muscle function is restored, and recognizing age-specific risk factors can help in making well informed decisions that reduce risk of graft rupture and secondary ACL injuries

Limitations

This review has several limitations that need to be acknowledged. First, the scarcity of studies available on this topic is a notable limitation. Additionally, the absence of studies presenting prolonged outcomes following pediatric ACL injury and reconstruction is evident, which limits a comprehensive understanding of the treatment’s lasting effects. Furthermore, we did not include conference abstracts and articles published in different languages with no available English translation, which may introduce a bias toward certain perspectives or findings. 

To enhance the comprehensiveness and reliability of insights, a focused systematic review and meta-analysis should be considered. Such an approach would potentially offer a more systematic and thorough examination of the topic, potentially yielding a higher level of evidence and a more robust understanding of the issues at hand.

## Conclusions

The findings of this study underscore the importance of early surgical reconstruction in children, driven by the desire for a swift RTS and the need to prevent the development of instability that can lead to progressive cartilage and meniscal damage. The emergence of various ACLR techniques that spare physis has facilitated favorable clinical outcomes while minimizing growth disturbances. Among the available autograft options, hamstring autografts are the preferred choice.

It is worth noting that complications such as graft rupture and contralateral ACL injuries are significantly more common following pediatric ACLR compared to adult ACLR, with rates being two to three times higher. In light of these findings, implementing neuromuscular training protocols is advised to mitigate the risk of complications such as graft ruptures. Overall, this study serves as a valuable reference for clinicians and researchers in the field, shedding light on the optimal strategies for managing pediatric ACL injuries and achieving successful outcomes.
